# Supplementary material for: Tead1a Initiates Transcriptional Priming Through the TEAD1a/YAP‐Notch1‐Spi1/Cebpα Axis to Promote Neutrophil Fate
Source: Adv Sci (Weinh). 2025 Aug 28;12(41):e05441. doi: 10.1002/advs.202505441 (PMC12591127; doi:10.1002/advs.202505441)
Supplement: Supplementary file 4 — Supplementary Dataset [file ADVS-12-e05441-s004.zip › 3-7.zip!/3-7/02-7.cluster/cluster18/Enrich/ego_all.pdf]

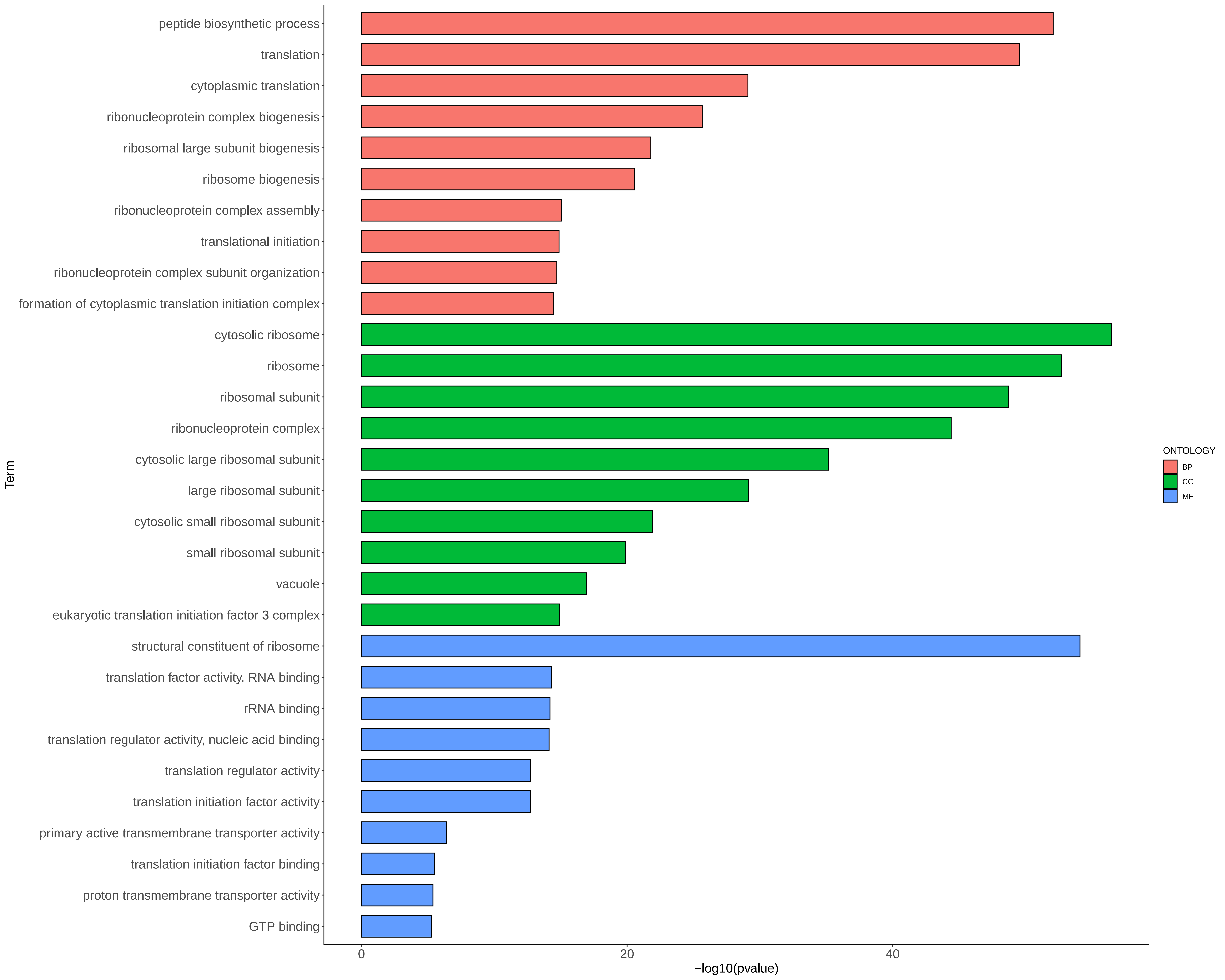
peptide biosynthetic process
translation
cytoplasmic translation
ribonucleoprotein complex biogenesis
ribosomal large subunit biogenesis
ribosome biogenesis
ribonucleoprotein complex assembly
translational initiation
ribonucleoprotein complex subunit organization
formation of cytoplasmic translation initiation complex
cytosolic ribosome
ribosome
ribosomal subunit
ribonucleoprotein complex
cytosolic large ribosomal subunit
large ribosomal subunit
cytosolic small ribosomal subunit
small ribosomal subunit
vacuole
eukaryotic translation initiation factor 3 complex
structural constituent of ribosome
translation factor activity, RNA binding
rRNA binding
translation regulator activity, nucleic acid binding
translation regulator activity
translation initiation factor activity
primary active transmembrane transporter activity
translation initiation factor binding
proton transmembrane transporter activity
GTP binding
Term
0
20
40
−log10(pvalue)
ONTOLOGY
BP
CC
MF
